# Adoption of conserved developmental genes in development and origin of the medusa body plan

**DOI:** 10.1186/s13227-015-0017-3

**Published:** 2015-05-29

**Authors:** Johanna E. M. Kraus, David Fredman, Wei Wang, Konstantin Khalturin, Ulrich Technau

**Affiliations:** Department for Molecular Evolution and Development, Centre for Organismal Systems Biology, University of Vienna, Althanstraße 14, Wien, Vienna 1090 Austria; Present address: Computational Biology Unit, University of Bergen, Thormohlensgate 55, 5008 Bergen, Norway; Zoologisches Institut, Christian-Albrechts Universität zu Kiel, Am Botanischen Garten 1-9, Kiel, 24118 Germany; Marine Genomics Unit, Okinawa Institute of Science and Technology, 1919-1 Tancha, Onna-son, Kunigami-gun, Okinawa 904-0495 Japan

**Keywords:** Life cycle, Polyp-medusa metagenesis, *Clytia hemisphaerica*, *Aurelia aurita*, Body plan evolution

## Abstract

**Background:**

The metagenesis of sessile polyps into pelagic medusae in cnidarians represents one of the most ancient complex life cycles in animals. Interestingly, scyphozoans and hydrozoans generate medusae by apparently fundamentally different processes. It is therefore unclear whether medusa formation has evolved independently in different medusozoans. To this end, a thorough understanding of the correspondence of polyp and medusa is required.

**Results:**

We monitored the expression patterns of conserved developmental genes in developing medusae of *Clytia hemisphaerica* (Hydrozoa) and *Aurelia aurita* (Scyphozoa) and found that developing medusae and polyps share similarities in their morphology and developmental gene expression. Unexpectedly, however, polyp tentacle marker genes were consistently expressed in the developing medusa bell, suggesting that the bell of medusae corresponds to modified and fused polyp tentacle anlagen.

**Conclusions:**

Our data represent the first comparative gene expression analysis of developing medusae in two representatives of Scyphozoa and Hydrozoa. The results challenge prevailing views about polyp medusa body plan homology. We propose that the evolution of a new life stage may be facilitated by the adoption of existing developmental genes.

**Electronic supplementary material:**

The online version of this article (doi:10.1186/s13227-015-0017-3) contains supplementary material, which is available to authorized users.

## Background

**Fig. 1:**
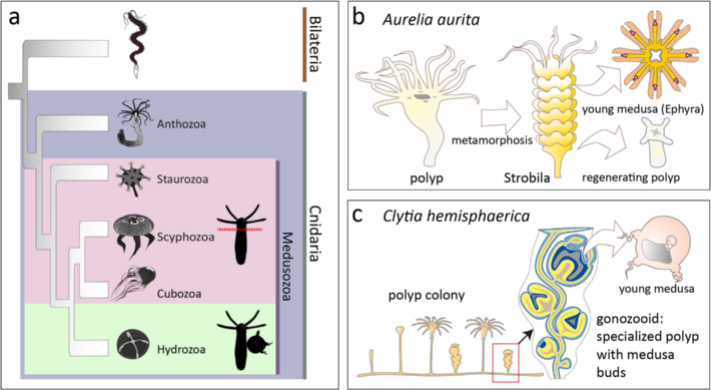
Principles underlying medusa formation in cnidarians. **a** Phylogenetic relationship of the cnidarian taxa (*blue box*) [14, 55, 56]. Anthozoans only form polyps, whereas medusozoan taxa show life cycles with polyps and medusae. Staurozoans, cubozoans and scyphozoans form their medusae by different forms of apical metamorphosis of polyps (*pink box*) [55]. Hydrozoans form their medusae through lateral budding on polyps (*green box*). **b** Polydisc strobilation in *Aurelia aurita*. **c** Lateral budding of young medusae in *Clytia hemisphaerica* from a specialized polyp form within the polyp colony

Complex life cycles involve a succession of life stages with drastically divergent body forms, behaviours and ecological habitat [1]. The emergence of complex life cycles is driven by the exploitation of different ecological niches and seasonally available resources [1]. The wide distribution of complex life cycles shows that such phenomena constitute an evolutionary advantage in virtually all eukaryotic phyla. Cnidaria, the sister group of the Bilateria, represent the oldest of all animal lineages with a complex life cycle. Cubozoa, Scyphozoa and Hydrozoa generally show a triphasic life cycle with a succession of a larva, a sessile polyp form and a pelagic, sexually active medusa stage (Fig. 1a). A polyp-like form has been suggested by many authors as the ancestral cnidarian adult body plan, and the medusa life stage a later inserted secondary derivative [2–8], although other scenarios have also been proposed (e.g. [9, 10]). The generation of medusae occurs in fundamentally different ways in different medusozoan taxa. A process called strobilation, where medusae form by apical metamorphosis of the polyp body followed by horizontal fission, is found in scyphozoans. Also in cubozoans, medusae are generated by apical metamorphosis from polyps [11] (Fig. 1b). While most authors interpret the cubozoan medusa formation as a complete metamorphosis from polyp to medusa [11, 12], others understand it as a modified form of strobilation [13]. Staurozoans are unusual as they form ‘stalked medusae’ by apical metamorphosis of the polyp but do not subsequently undergo fission [14, 15]. In contrast to the three other medusozoan taxa, medusae of hydrozoans (‘hydromedusae’) are generally formed in a lateral budding process from polyps. Uniquely, this process involves the generation of a third tissue layer in between endo- and ectoderm, the so-called entocodon, previously proposed as a potential homologous layer to the bilaterian mesoderm [9, 10] (Fig. 1c).

In summary, apical metamorphosis of polyps and lateral budding represent two fundamental principles of medusa formation. To uncover the evolution of the cnidarian metagenesis, it is first necessary to understand how the body plans of polyp and medusa relate to each other. This is a longstanding debate for over 150 years [2, 7, 16–21]. In particular, it was disputed which part of the polyp corresponds to the medusa bell, the most distinctive feature of jellyfish forms [16, 19–21]. In many zoology textbooks, the adult medusa body plan is depicted as a polyp turned upside-down with an enlarged peristomial region and an extremely shortened oral-aboral axis [7, 22]. However, this comparison is primarily based on morphological similarities of adult forms. Divergent from this view, Allman and Hadzi, based on their studies of hydrozoan medusa formation, suggested that the medusa bell is derived from polyp tentacle anlagen fused to each other by an enlarged hypostome [19, 21]. A largely neglected hypothesis was put forward by Huxley, who interpreted the elongated mouth tube of hydromedusae together with oral tentacles, present in some species, as representing the entire body plan of the polyp [16]. He named this the “polypite” and interpreted the medusa bell as a later added organ for swimming without a clear homolog to the polyp body plan [16]. Similar to this, Metchnikoff also saw the polyp body represented in the mouth tube of hydromedusae; however, he interpreted the medusa bell as a modified web of stolons of previously colonial forms [20].

Here, by comparing gene expression patterns during different developmental stages, we suggest that the medusa bell is formed from modified polyp tentacle anlagen, while the polyp hypostome corresponds to the medusa manubrium. This challenges the prevailing view of medusa and polyp body plan correspondence and suggests a scenario for the emergence of another adult life stage.

## Methods

*Clytia hemisphaerica* and *Aurelia aurita* were cultured as previously described [23].

### Library preparation and cloning of genes

Transcriptome libraries were created with high quality total RNA (RQI values ranging between 8 and 10) of a single juvenile jellyfish (*Aurelia*) and several adult medusae (*Clytia*). Following mRNA purification by poly-A selection, cDNA library preparation was done using reagents provided by the TruSeq® RNA Sample Preparation Kit v2 (Illumina®) according to the manufacturer’s protocol with two changes: (a) fragmentation of the mRNA at 80 °C for 3 min and (b) using half volumes of PE Adapter Oligo Mix in the adapter ligation step. The libraries were paired-end sequenced on the Illumina HiSeq 2000 platform, with a 100-bp read length to a total depth of 216–220 million reads. Reads of low quality, low complexity, containing adapter sequence or matching ribosomal or mitochondrial sequence were discarded. The library insert size used for assembly was estimated by mapping a subsample of reads to reference transcripts. Transcriptomes were assembled using Oases [24] with multiple k-mers in the range 53–81 and Trinity [25] with default settings. The resulting transcripts were merged into unigenes using TGICL [26], and transcripts with good matches to food sources (*Artemia salina*) or bacteria were removed. The resulting *Clytia* transcriptome covered 67.6 Mb in 39,979 transcripts, with a median length of 1.3 kb, mean of 1.7 kb and N50 of 3.9 kb. The resulting *Aurelia* transcriptome covered 89 Mb in 81,158 transcripts, with a median length of 0.8 kb, mean of 1.1 kb and N50 of 2.5 kb. The sequence data and transcriptome assemblies are deposited in the NCBI TSA archive.

### In situ hybridization

*Aa-gata*, *otx* and *bmp5/8* on strobilae and polyps were performed as previously described [27]. All other *Aurelia* and *Clytia* in situ hybridization experiments were done according to [28], with some modifications. A bleaching step in 0.5 % H_2_O_2_/5 % formamide/0.5× saline sodium citrate (SSC) in H_2_O for 5 min at room temperature (RT) was added after rehydration. Proteinase K digest was done for 20 min in 1 μg/ml Proteinase K (Ambion) in 1× PBS with 0.2 % Tween 20 (Sigma-Aldrich) at RT. Three percent Blocking reagent (Roche) and 5 % dextran sulphate (Sigma) were added to the hybridization mix. The samples were incubated in the hybridization mix over night without probe at hybridization temperature (58 °C) and subsequently hybridized for 36 h with 0.25 ng/μl digoxigenin (DIG)-labelled RNA probe. After hybridization, the samples were gradually transferred to 2× SSC at 58 °C. Subsequently, they were incubated for 40 min in 1 U/μl RNAse T1/2× SSC at 37 °C, followed by 3 × 20 min washes in 0.2× SSC at 58 °C to reduce unspecific staining. Next, the samples were transferred to maleic acid buffer (MAB) at room temperature and blocked for 1–2 h in 1 % Blocking reagent (Roche) in MAB. The samples were then incubated in 1:2000 anti-DIG antibody (Roche) in a blocking solution overnight at 4 °C. Subsequently, the samples were transferred to 1× PBS with 0.1 % Triton X-100 (PTx) and after extensive washes, stained according to [28].

### F-actin and nuclear staining of *C. hemisphaerica*

Young medusae, gastrozooid polyps and gonozooids were fixed overnight in 4 % paraformaldehyde at 4 °C. Thecae of gonozooids were removed mechanically before fixation. Animals were subsequently washed in PTx and incubated in 3 μl/100 μl Phalloidin Alexa 488/1:1000 DAPI/5 % sheep serum/PTx overnight at 4 °C in the dark. After extensive washes in PTx at 4 °C, the samples were mounted in Vectashield mounting medium for fluorescence (Vector).

### Phylogenetic gene trees

Sequence alignments and neighbour-joining trees were calculated using the built-in algorithm of Clustal X [29] (Additional files 1, 2, 3, 4, 5, 6 and 7). Sequences were trimmed using Gblocks [30]. Best models for maximum-likelihood trees were found with ProtTest3 [31]. Maximum-likelihood trees were calculated with PhyML [32]. Any newly investigated medusozoan gene was considered to be an ortholog of a given bilaterian gene family if supported in both maximum-likelihood and neighbour-joining phylogenetic trees.

## Results

### Early stages of hydrozoan medusae morphologically resemble polyp buds

**Fig. 2:**
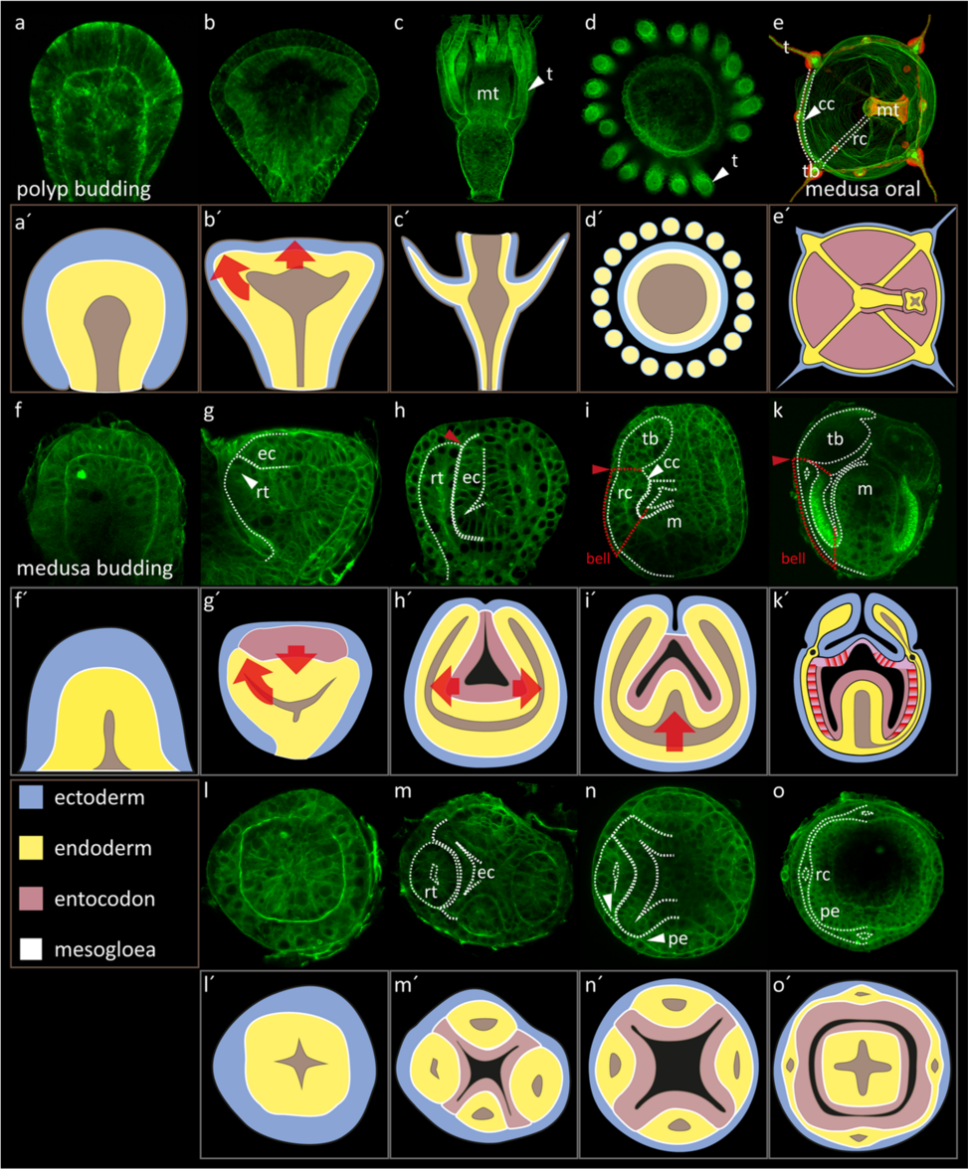
Initial processes during *Clytia* medusa formation show parallels to polyp bud development. F-actin staining, single confocal sections. Earlier stages *left*, later stages *right*. **a**–**d**’ Polyp budding, *mt* mouth tube, *t* tentacle, **d**, **d**’ oral view of an adult polyp. **f**–**o**’ Medusa budding. **e**, **e**’ Oral view of a young medusa, *stippled lines* outline the canals of the gastro-vascular system. *cc* circular canal, *tb* tentacle bulb, *rc* radial canal, *mt* mouth tube. *Red* nuclei (TOPRO3). **f**–**k**’ Lateral views, **l**–**o**’ oral views. *Stippled lines* demarcate the ECM between the tissue layers **h**–**k**, **m**–**o**. The endoderm forms radial tubes (*rt*) as the entocodon (*ec*) sinks inwards **g**, **g**’ *arrows*. The radial tubes are covered by a layer of outer ectoderm externally and a layer of entocodon tissue internally **h**, **h**’, **m**, **m**’. *Red arrowhead* **h** Beginning of formation of the velar plate demarcates the future bell rim. **i**, **i**’, **k**, **k**’ The distal halves of the radial tubes develop into medusa tentacle bulb endoderm (*tb*). The radial tube portion up to this level gives rise to the radial (*rc*) and circular canals (*cc*) of the gastro-vascular system and the plate endoderm (*pe*) of the bell (*stippled red lines*). The outgrowth of the circular canals (*cc*, *white arrowhead*) starts at the level of the future bell rim (*red arrowhead*). The mouth tube (*m*) starts to grow into the entocodon-cavity. **n**–**o**’ Oral views of representative buds reveal the formation of the umbrellar plate endoderm from lateral outgrowth and fusion the four radial tubes (*white arrowheads*). (**o**, **o**’) Late medusa bud with completed plate endoderm and radial canals

In order to compare polyp and medusa development in the hydrozoan model species *C. hemisphaerica*, we monitored the morphogenesis of medusa formation using F-actin staining (Fig. 2). In *Clytia*, as typical for colonial hydrozoans [17, 19, 33, 34], both polyps and medusae are formed by lateral budding processes. The development of a polyp bud is characterized by three key events: the budding process begins with an outgrowth of ecto- and endoderm (Fig. 2a, a’). Subsequently, tentacles start to form distally on the polyp bud (Fig. 2b, b’). Polyp formation is completed by the outgrowth of tentacles and mouth tube and finally by the breakthrough of the mouth opening (Fig. 2c, c’, d, d’).

We found that medusa development in *Clytia* is characterized by similar events during early budding stages (Fig. 2f–h, f’–h’). Medusa formation also begins with the bulging out of ecto- and endoderm from the body wall of the mother polyp. A group of cells delaminates from the distal ectoderm, forming the entocodon, which displaces the bud endoderm and later forms the mouth tube ectoderm and the lining of the subumbrella (Fig. 2g–o’). The remaining bud ectoderm forms the entire lining of the exumbrella, the outer lining of the velum and the tentacle ectoderm (Fig. 2k, k’). The endoderm develops into the entire gastro-vascular system of the bell and the inner medusa tentacle epithelium by a process involving two major morphogenetic events. First, the initially homogenous endoderm forms four hollow radial tubes that lie in between the surface ectoderm and the entocodon (Fig. 2h, h’, m, m’). Notably, the distal halves of the tubes develop into the medusa tentacle endoderm, while the proximal halves develop into the plate endoderm, the circular canal and the four radial canals of the medusa bell by a process that appears to involve a lateral fusion of epithelia (Fig. 2n, n’, o, o’). Thus, early medusa development in hydrozoans resembles polyp budding.

In contrast to hydrozoans, scyphozoans like *A. aurita* typically generate medusae by polydisc strobilation [35] (Fig. 1b). Strobilation is initiated by the formation of numerous evenly spaced constrictions along the entire length of the polyp body, which gradually deepen and subdivide the polyp into a stack of discs. Each disc then grows out eight so-called rhopalar arms, a process reminiscent of tentacle formation in polyps, and develops into a juvenile medusa, a so-called ephyra. The mouth of the ephyra, which appears relatively late in development, is always oriented towards the oral end of the original polyp. Prior to their detachment, the individual ephyrae start to rhythmically contract their rhopalar arms until they are released into the surrounding water.

### Polyp oral marker genes are restricted to oral regions in medusae

**Fig. 3:**
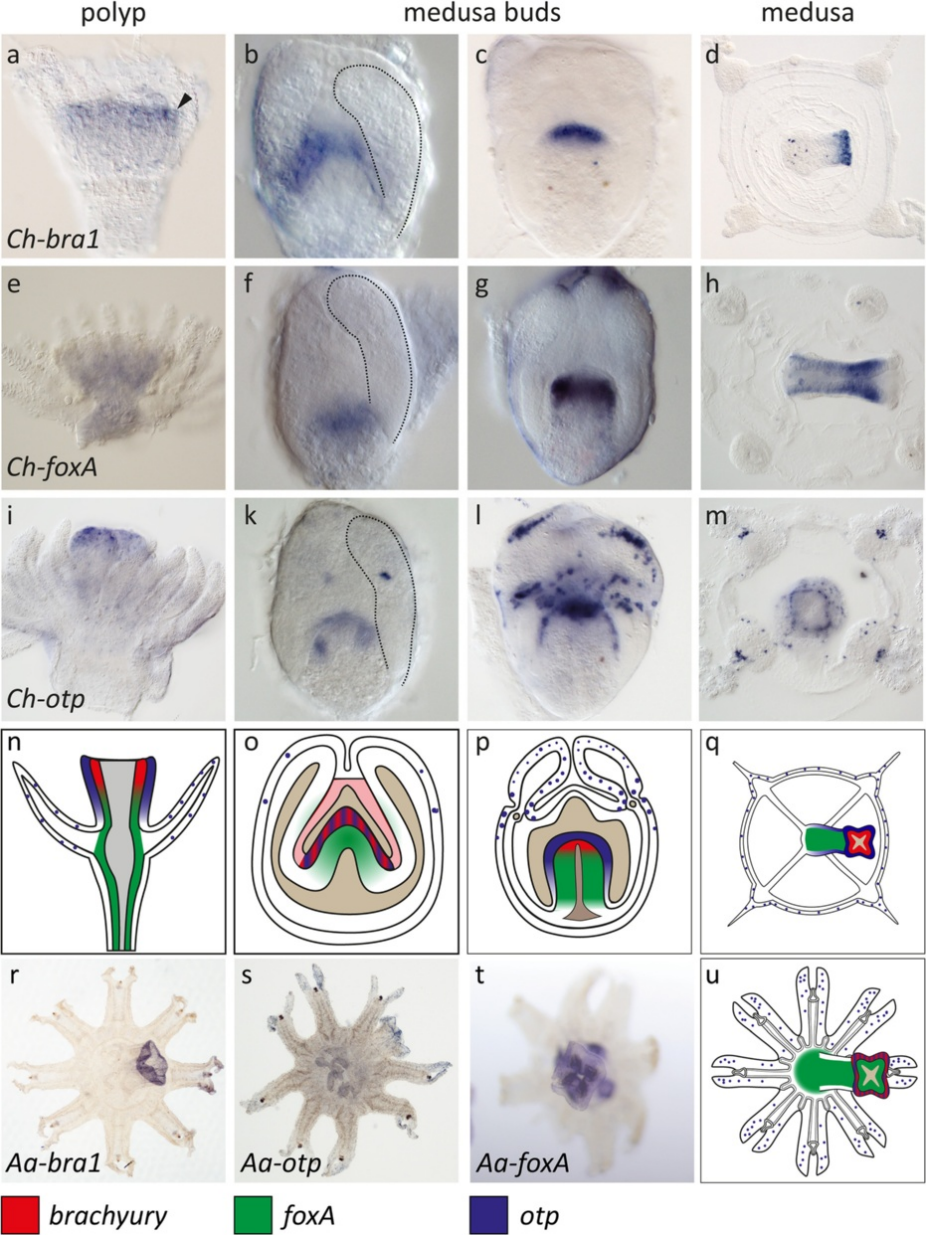
Oral marker gene expression is mostly restricted to developing oral regions in polyps and medusae of Clytia hemisphaerica and Aurelia aurita. (**a**) Expression of *Ch-bra1* in the mouth endoderm. (**b**–**d**) During medusa formation, it is expressed from the earliest budding stages onwards, initially in the entire entocodon, then restricted to the oral ectoderm. In late medusa buds and the medusa, *Ch-bra1* is restricted to the mouth endoderm. (**e**) *Ch-foxA* expression in the entire head endoderm in polyps but absent from the tentacles. (**f**–**h**). During medusa formation, the onset of *Ch-foxA* expression coincides with the appearance of the mouth tube. In later medusa buds and in the free medusa, the entire mouth tube apart from the mouth tip region is *foxA*-positive. (**i**) *Ch-otp* expression in the polyp ectodermal mouth region and in single cells in the tentacles. In early medusa budding stages (**k**), *Ch-otp* is expressed in cell clusters in the developing oral ectoderm and in single cells in the outer bud ectoderm. In late medusa budding stages and medusa the oral expression of otp becomes more prominent and numerous *otp*-positive cells and cell clusters appear at the bell rim and at the aboral side of the tentacle bulbs and single cells at the bell rim. (**n**–**q**) Schematics illustrate oral marker gene expression in the representative stages of *Clytia*. (**r**) *Aurelia* ephyra show *Aa-bra1* expression in the mouth tip. s *Aaotp* expression in single cells of the rhopalar arm ectoderm and the mouth tip. (**t**) *AafoxA* expression in the entire mouth tube of the ephyra but not in the lips (**u**) Schematic shows summary of mouth marker expression in the *Aurelia* ephyra

The current model of polyp-medusa body plan homology assumes that the polyp mouth region corresponds to the entire subumbrella of medusae [7, 22, 36]. If correct, this model implies that the expression of conserved polyp mouth marker genes should expand to future subumbrellar regions during medusa formation (Fig. 1d). We tested this hypothesis by comparing the expression of *Clytia* and *Aurelia* orthologs of the T-box gene *brachyury* (*bra*), the winged helix gene *forkhead-box transcription factor A* (*foxA*) and the homeobox gene *orthopedia* (*otp*) between developing polyps and medusae. These transcription factors were chosen for their conserved oral (or blastoporal) expression domains in the anthozoan *Nematostella vectensis* [37–39] and in bilaterians [40–42]. Accordingly, these genes were all expressed specifically in an oral domain of the *Clytia* polyp (Fig. 3a, e, i, n). In contrast to the prediction, however, we found that *brachyury* and *foxA* orthologs were never expanded to developing bell regions during the development of *Clytia* medusae or *Aurelia* ephyrae but were restricted to oral regions in both species (Fig. 3a–u). Both *Ch-otp* and *Aa-otp* were expressed not only in the oral ectoderm but also in single ectodermal cells in the tentacles of the *Clytia* polyp, in the developing bell rim and tentacles of *Clytia* medusae and in the rhopalar arms of the *Aurelia* ephyra. It is possible that these cells belong to the neuronal lineage, given that *otp* marks neurons in many bilaterians [43]. Together, these findings indicate that the polyp mouth region and the medusa bell do not share a common profile of hallmark transcription factor expression during their development and might thus not share a common evolutionary and developmental origin.

### Endodermal polyp tentacle marker genes are expressed in the bell endoderm of medusae

**Fig. 4:**
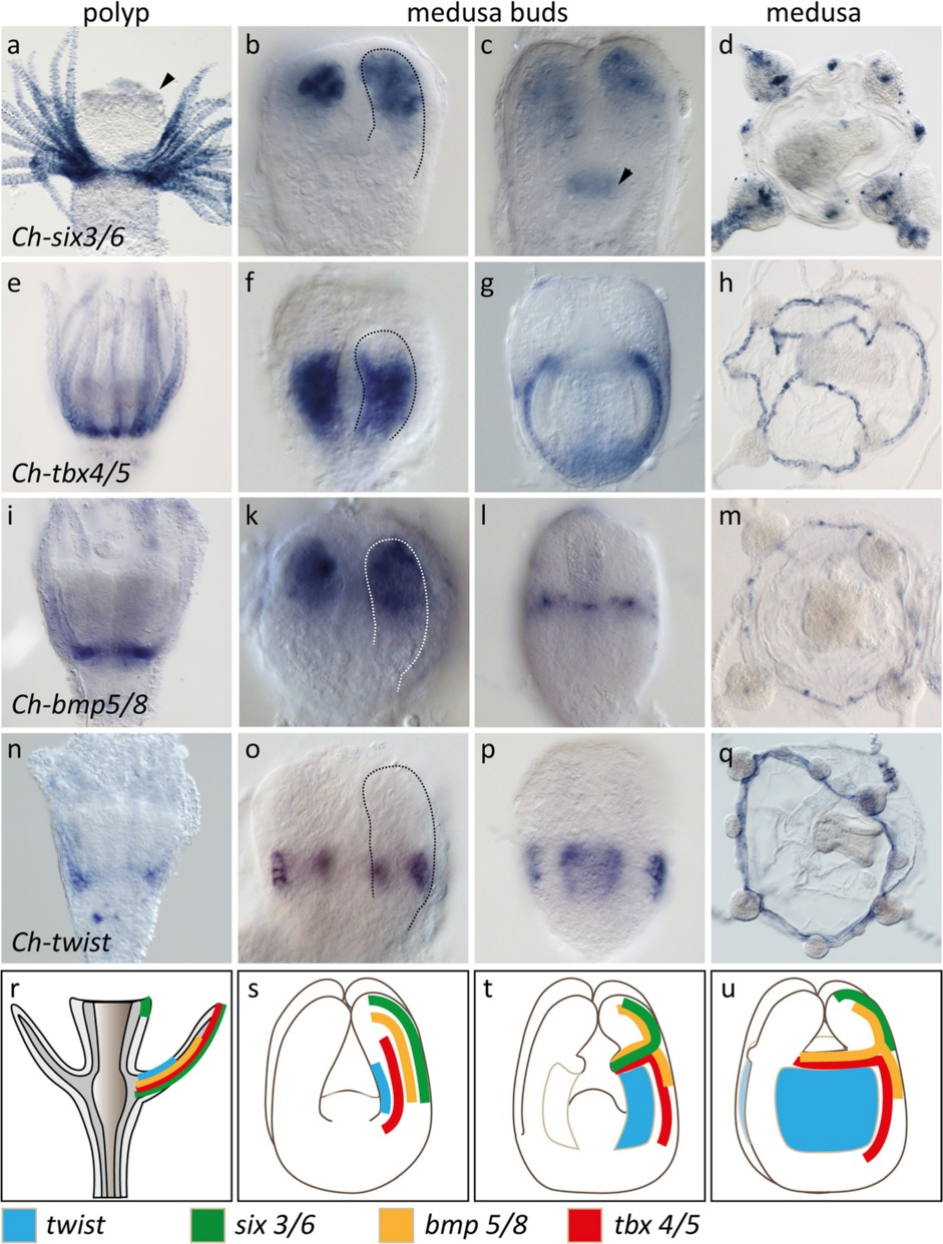
Endodermal polyp tentacle marker in the developing bell endoderm of *Clytia* supports a homology of radial tubes and the bell endoderm. (**a**) *Ch-six3/6* expression in the tentacle endoderm of polyps, and additional weak staining of the mouth tip ectoderm. (**b**–**d**) *Ch-six3/6* expression in the radial tubes and in later medusa buds also in the ectoderm of the mouth anlage (arrowhead). In the young medusa, *Ch-six3/6* is found in cell clusters on the tentacles and the bell rim. (**e**) *Ch-tbx4/5* expression in polyp tentacle endoderm. (**f**–**h**) In the medusa bud, *Ch-tbx4/5* expression is restricted to the basal ‘bell part’ of the radial tubes (dotted line). In young medusa *Ch-tbx4/5* remains expressed in the endoderm of the future canals of the gastro-vascular system. (**i**) *Ch-bmp5/8* expression at the base of the polyp tentacles. (**k**–**m**) During medusa formation, *Ch-bmp5/8* is expressed in the radial tubes at early stages. in young ephyrae *Ch-bmp5/8* is expressed in sensory structures at the bell rim. (**n**) In polyps, Ch-twist is expressed in the polyp tentacle endoderm, strongest at the base. (**o**–**q**) During medusa formation, the expression of *Ch-twist* is specifically expressed in the laterally outgrowing cells from each side of the bell part in the radial tubes, initially within the *Ch-tbx4/5* domain (see **f**), subsequently in the whole umbrellar plate endoderm. In the medusa stage, *Ch-twist* is expressed at a low level in the plate endoderm but more strongly at the bell rim. **r**–**u** Schematic summary of the expression patterns. **r** Polyp. **s**–**u** Early, intermediate and late medusa bud. Only endodermal tissues shown for clarity

\An alternative and largely neglected view of polyp and medusa body plan homology suggests that the medusa bell is derived from fused polyp tentacle anlagen [19, 21]. This model predicts that polyp tentacle endoderm corresponds to the radial tubes, while polyp tentacle ectoderm would be related to the bell ectoderm. We tested this hypothesis by studying the expression of polyp tentacle endoderm marker genes in the developing medusa. We found that *twist*, *tbx4/5*, *bmp5/8* and *six3/6* were specifically expressed in tentacles of the *Clytia* polyp (Fig. 4a). In addition, *six3/6* expression was also detected at the mouth tip. When analysing the expression of these genes during medusa development in *Clytia*, we found that all genes were expressed in different parts of the radial tubes in the medusa buds (Fig. 4). The spatial expression of these genes gradually changed during differentiation and was in fully differentiated medusae restricted to the tentacle bulb ectoderm (*six3/6*), canal endoderm (*tbx4/5*, *bmp5/8*), statocysts (*bmp5/8*) and the bell rim endoderm (*twist*). *Six3/6* has previously been investigated in adult medusae of *Podocoryne carnea* and *Cladonema radiatum*, where it showed a fairly similar expression in the tentacle bulbs [44]. Supporting our findings in *Clytia*, the expression of a *twist* and a *bmp 5/8* homolog was previously reported in *P. carnea* during medusa formation with very similar expression domains in the developing medusa bell [45, 46].

**Fig. 5:**
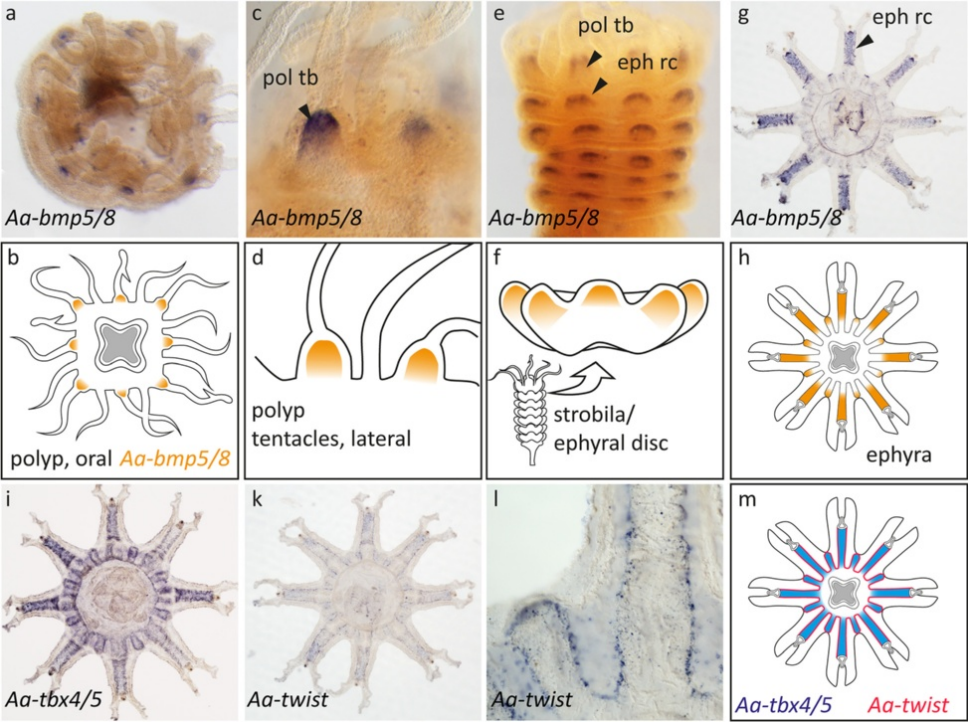
The rhopalar arm endoderm of *Aurelia* ephyrae shares a set of marker genes with *Clytia* radial tubes. **a**, **b** Oral view on a polyp. *Aa-bmp5/8* expression in eight tentacle bases. **c**, **d** Lateral view on the polyp tentacles (*arrowhead*, *pol tb*. **e**, **f**) Young strobila during outgrowth of the ephyral rhopalar arms. *Bmp-5/8* expression both in the remaining polyp tentacle bulbs (*arrowhead*) and the ephyral radial canals (*arrowhead*, *eph rc*). **g**, **h** Free ephyra. The radial canals of the eight primary rhopalar arms are *Aa-bmp5/8*-positive, as well as the outgrowing velar arms. **i**, **m** Broad expression of *Aa-tbx4/5* in the rhopalar arms and velar arm endoderm. **k**–**m** *Aa-twist* is mainly expressed in a row of cells lining the canals of the gastro-vascular system. **l** detail of **k**, showing additional *twist*-positive cells in the endoderm between the canals

These data strongly support the hypothesis that the radial tubes, developing basally into the bell gastro-vascular system and distally into the endoderm of the medusa tentacles, share a common origin with the polyp tentacle endoderm. Strikingly, despite the fundamentally different mode of medusa formation by strobilation, this view is further supported by analysis of marker gene expression in *Aurelia. Aa-bmp5/8* expression, for example, reflects the formation of ephyral rhopalar arms from the eight primary polyp tentacles (Fig.5a–h). In addition, *Aa-twist* and *Aa-tbx4/5* orthologs were expressed in the endodermal radial canals of the rhopalar arms and the velar arms that occupy analogous positions to the radial tubes of *Clytia* medusa buds. More specifically, in both *Aurelia* and *Clytia*, *twist* was expressed in a row of cells lining the radial canals or radial tubes, respectively (compare Fig. 4o with Fig. 5l). Endodermal tentacle markers thus further support a homology of the bell and polyp tentacles in both scypho- and hydromedusae.

### The bell ectoderm expresses ectodermal polyp tentacle markers

**Fig. 6:**
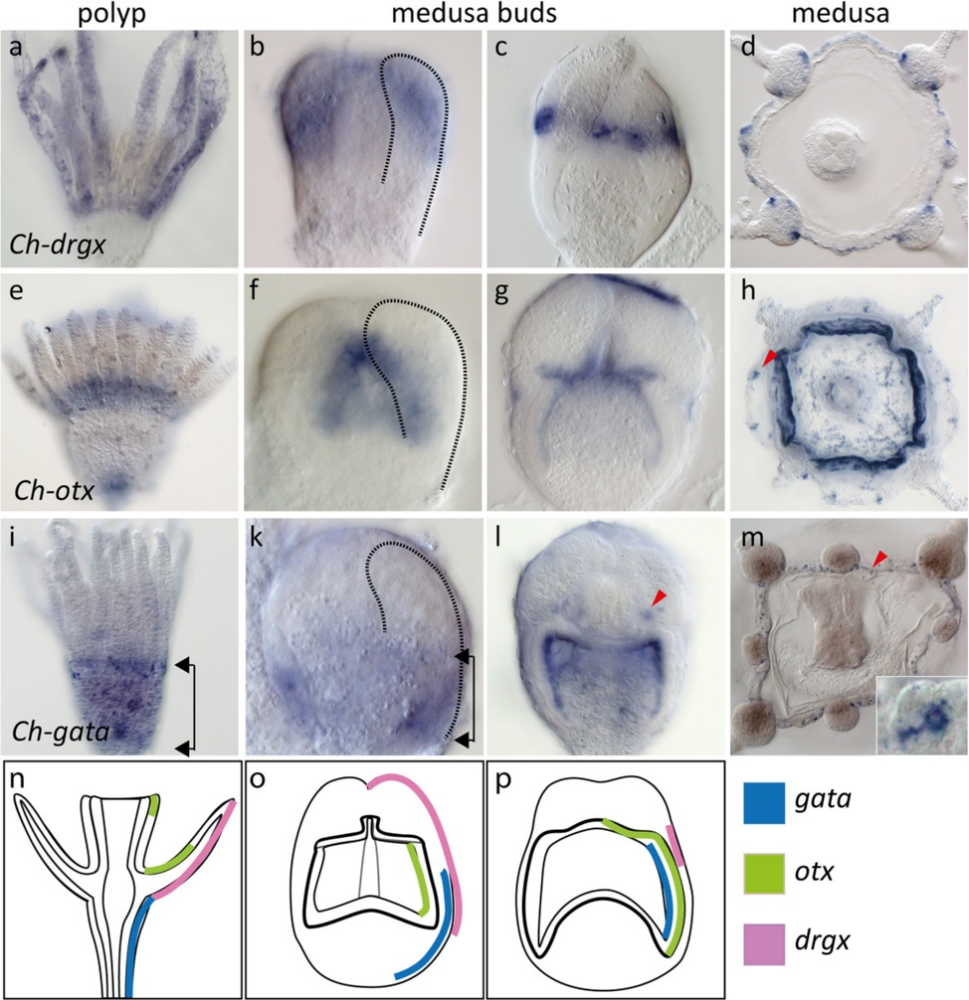
Expression of *drgx*, *otx* and *gata* in *Clytia*. (**a**–**d**) *Ch-drgx* expression in the tentacle ectoderm of polyps. (**b**–**d**). In early medusa buds, it is expressed in the ectoderm covering the radial tubes (stippled line) externally. In the late medusa bud, *Ch-drgx* is expressed in a narrow band around the future bell rim. In free medusa, *Chdrgx* is expressed at the bell rim. (**e**–**h**) *Chotx* is expressed in polyps at the tentacle base ectoderm and in the mouth tip. In early medusa buds, *Ch-otx* is expressed in the lateral parts of the entocodon (**f**, stippled line marks radial tube). In late medusa buds, *Ch-otx* expression is located in the entire ectodermal subumbrella with strongest expression in the inner bell rim but absent from the striated muscle-differentiating areas. In young medusa *Ch-otx* is found in diffuse cell clusters in the entire subumbrella and in statocysts (red arrowhead). (**i**–**m**) *Ch-gata* is expressed in the body column ectoderm up to the tentacle bases of the polyp (arrowheads). Early medusa bud express *Ch-gata* in the basal outer ectoderm below the level of the radial tubes (arrowheads). Late medusa buds show *Ch-gata* expression in the entire subumbrellar ectoderm and weaker staining in the exumbrellar ectoderm but absence of *Ch-gata* signal in the striated muscle sheet. Additional *Ch-gata* expression in the developing nerve rings at the bell rim (red arrowhead). In free medusae, *Ch-gata* is exclusively expressed in single cells at the bell rim, presumably in neurons (**m**, red arrowhead, insert). (**n**–**p**) Schematic summary of expression patterns of ectodermal polyp tentacle marker genes (right halves) and general morphology (left halves) in polyps, early medusa buds and late medusa buds. Only ectodermal tissue is shown for better clarity

In order to test the alternative scenario of polyp-medusa homology, we asked if not only endodermal but also ectodermal polyp tentacle marker genes are expressed in the medusa bell during development. Indeed, the genes *drgx* and *otx*, consistently expressed in the ectoderm of polyp tentacles, were found in various areas of the forming medusa bell ectoderm in *Clytia*, adding further support to the hypothesis that the medusa bell and polyp tentacles are homologous structures (Fig. 6).

*Ch-drgx* is expressed over the entire length of the polyp tentacle ectoderm (Fig. 6a). During early stages of medusa formation, the outer ectoderm covering the radial tubes was found to be positive for *Ch-drgx* expression (Fig. 6b). During plate endoderm and circular canal formation (Fig. 6c), the expression of *drgx* was gradually restricted to a narrow band around the future bell rim and the forming medusa tentacles and is still found expressed at the same location in young medusae (Fig. 6d).

*Clytia otx* was expressed in young polyp stages in the tentacle base ectoderm and at the tip of the mouth (Fig. 6e). In addition, it was detected in individual cells and cell clusters in the tentacle ectoderm, whose cell shape indicate that they may be sensory neurons. During medusa development, *Ch-otx* expression was restricted to entocodon cells covering the striated muscle sheet (Fig. 6g). In free medusae, *Ch-otx* was expressed in numerous cells not only in the subumbrella but also in the bell rim, where it is presumably expressed in neurons of the nerve ring, including the sensory organs, and the statocysts (Fig. 6f, red arrowhead).

In *Clytia* polyps, *Ch-gata* was expressed in the body column ectoderm up to the tentacle bases (Fig. 6i, black arrowhead), like shown for *Hydra* [47]. During medusa formation, it was expressed in the basal half of the ectoderm from the very earliest stages onwards (Fig. 6k). Later, it showed an additional expression domain in the entocodon-derived inner ectoderm, similar to *otx*, (Fig. 6l). However, *Ch-gata* was expressed in cells covering the muscle cell layer and in cells of the two nerve rings, where it was also detected at later stages (Fig. 6 l, m, red arrowheads). Thus, *otx* and *drgx* shared overlapping expression domains in the ectoderm of polyp tentacles. In the developing medusa bell, they were expressed in different ectodermal domains surrounding the radial tubes: *drgx* was expressed along the outer surface of the radial tubes, while *otx* was expressed in the entocodon layer. Reminiscent of the polyp expression, *Ch-gata* marked early the aboral ectoderm up to the level of the radial tubes in medusa buds.

**Fig. 7:**
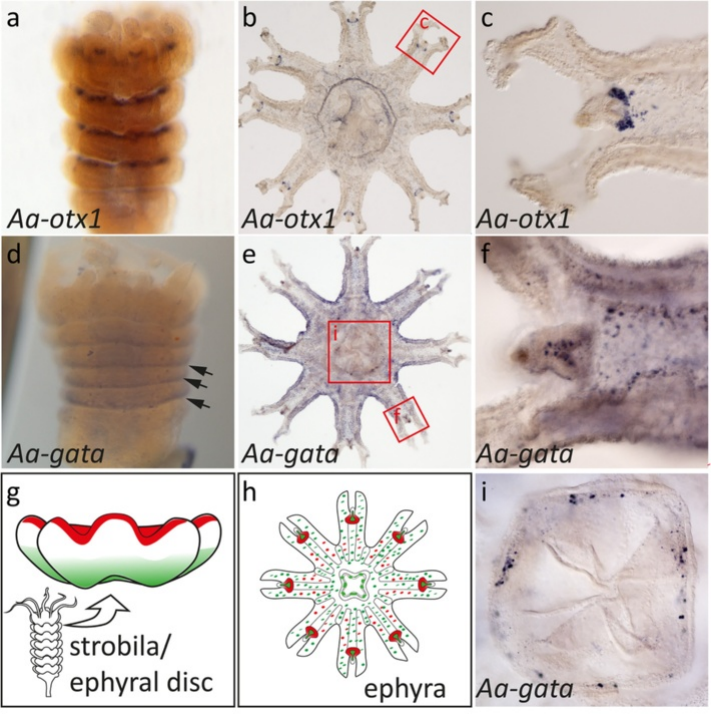
Expression of *Aurelia aurita otx* and *gata* orthologs during strobilation and in free ephyrae. **a**–**c** *Aa-otx1* is expressed in the oral ectoderm of each individual ephyral disc (**a**), before the *otx* signal gradually fades in later stages. *Aa-otx1* remains specifically expressed in individual cells at the oral ectoderm of the free ephyra (**b**) and in distinct cell clusters in the rhopalia (**c**). *Aa-gata* (**d**–**i**) is weakly expressed in the aboral ectoderm of each ephyral disc during strobilation (**d**, *arrowheads*). In the free ephyra, it is broadly expressed in the ecto-and endoderm (**e**, **f**) and in specific cells lining the mouth of the ephyra, presumably neurons (**h**, **i**). Colours in schematics represent *Aa-gata* (*green*) and *Aa-otx* (*red*). For clarity, broad *Aa-gata* expression and single cells of *Aa-otx* in the ephyral ectoderm are not represented in (**h**)

Given the completely diverging ways of medusa formation, the expression profiles of *gata* and *otx* orthologs in *Aurelia* strobilae were surprisingly similar to those in *Clytia* during early medusa budding stages. So far, *Aurelia otx* expression has only been studied in later ephyra stages, where it is restricted to the rhopalia, the compound sensory organs of scyphozoan jellyfish [48]. We found that during strobilation, *Aa-otx* was expressed in the oral side ectoderm of each ephyral disc from the earliest stages, where it is likely involved in setting up the motor nerve net, as previously suggested [48]. Later, the *otx* expression domain was gradually restricted to the rhopalia of the ephyra. A second *Aa-otx* gene was expressed in similar regions at levels near the detection limit (data not shown) (Fig. 7a–c).

*Aa-gata* was expressed in early strobilae at the aboral side of each ephyral disc (Fig. 7d, g), reminiscent of the aboral expression of *Ch-gata* in the polyp and early medusa buds. The *Aurelia* ephyrae expressed *gata* more broadly in the entire ectoderm, similar to intermediate stages of *Clytia* medusa bud. In addition, some localized expression in cells, presumably neurons, was detected at the mouth tip (Fig. 7h, i). We conclude, that *Clytia* and *Aurelia* share expression profiles of positional marker genes during development.

## Discussion

While a recent study shed some light on the molecular control of the induction of strobilation [27], virtually nothing is known about the developmental and genetic basis that characterizes the transition between polyp and medusa. It is also not clear whether the same rules apply to scyphozoans and hydrozoans with their drastically different modes of medusa formation.

**Fig. 8:**
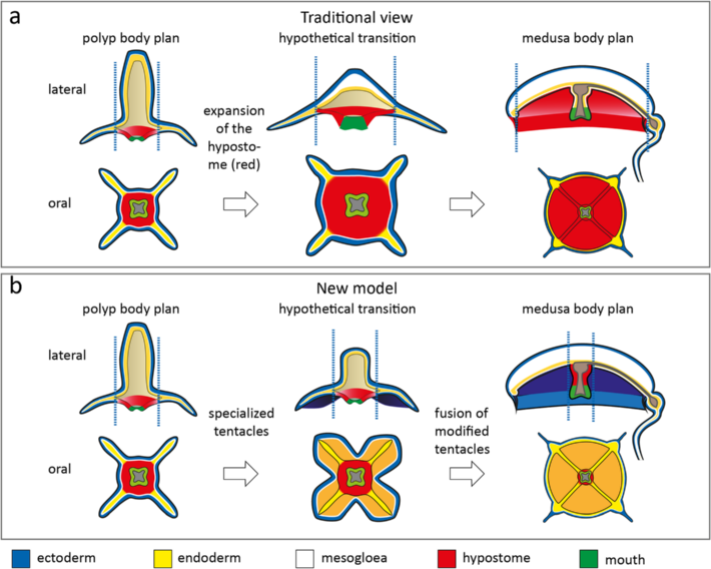
Prevailing view compared to new interpretation of the medusa body plan. **a** Prevailing view of polyp-medusa body plans: the medusa subumbrella corresponds to the hypostomal field of the polyp, and hence, the medusa represents a polyp with extremely shortened oral-aboral axis and an enlarged hypostome (*red*, corresponding colours). Upper row modified after [22]. **b** Proposed new model: the medusa bell is largely the product of fused tentacle anlagen

In this study, we compared conserved marker gene expression patterns in a series of developmental stages in the hydrozoan *Clytia* and the scyphozoan *Aurelia*, representing two major modes of medusa formation. We found both species polyp tentacle marker genes to be specifically expressed in the developing medusa bell. We propose that the bell is the evolutionary result of fusion processes of ancestral polyp tentacle anlagen, as suggested by Allman and Hadzi [19, 21], with the only difference that we find no evidence that the modification of the tentacle anlagen was accompanied by an enlargement of the polyp hypostome (Fig. 8b).

In line with this, the ectodermally derived entocodon, which transiently arises during medusa formation in hydrozoans, expresses ectodermal manubrium marker genes and tentacle marker genes in inner and lateral regions, respectively. This contradicts an earlier interpretation that the entocodon is homologous to the bilaterian mesoderm layer [9, 10, 45, 49] and the interpretation of the medusa being basically an oral-aborally flattened polyp form with an enlarged hypostome [7, 22] (Fig. 8a). Our gene expression data also contradict the hypothesis of a representation of the whole polyp body plan in the elongated mouth tube of the medusa [16, 20].

We propose that medusa formation is initiated by a polyp-like developmental programme, reflected by a similar early morphology of polyp and medusa buds in *Clytia* and by the deployment of genes marking various regions in the polyp. Notably, most of the investigated genes also have a conserved expression pattern in several other hydrozoans [45, 46, 50–53] as well as in the anthozoan *Nematostella* [37–39, 54], suggesting deeply conserved developmental roles in the cnidarians. Later in development of the medusa, as the morphological similarity of body plans of the medusa bud to a polyp gradually blurs, expression patterns accordingly diverge in the developing medusa.

Despite the strikingly different mechanisms of medusa formation in hydrozoans and scyphozoans, all marker genes investigated in *Aurelia* also showed corresponding expression patterns during strobilation. We propose that the ephyral rhopalar arms of *Aurelia* correspond to the developing bell of *Clytia*. Both structures express polyp tentacle markers and lack the expression of oral marker genes. These findings are consistent with the view that medusa formation evolved only once in the common ancestor of hydrozoans and scyphozoans, possibly of all medusozoans. Hence, we expect that these patterns might be shared more broadly among other medusozoan species. However, Cnidaria is an ancient animal lineage and the extant taxa display a stunning diversity of different polyp and medusa forms. In particular, further investigations in Cubozoa and Staurozoa will be needed to assess whether the same principles apply to medusa formation processes encountered in these groups. Moreover, since only limited expression patterns of conserved marker genes are available and functional data are lacking to date, it is conceivable that in other medusozoan lineages, other regulatory genes are crucial or that the same conserved genes became recruited independently to the process of medusa formation. Therefore, albeit at this point less likely, it cannot be ruled out that the medusa stage evolved several times independently from polyp forms in different medusozoan lineages [5]. The data presented here may stimulate more research aimed at a mechanistic understanding of medusa formation in various medusozoan representatives in order to reveal the changes in the developmental processes that led to the evolution of the medusa as an additional life stage.

## Conclusions

Our data represent the first comparative gene expression analysis of developing medusae in two representatives of Scyphozoa and Hydrozoa. The results challenge prevailing views about polyp medusa body plan homology. We propose that the evolution of a new life stage may be facilitated by the adoption of existing developmental genes.

## Additional files

Additional file 1:
**Phylogenetic analysis of Six homeobox transcription factors.** Maximum-likelihood and neighbour-joining analysis support orthology of cnidarian Six3 proteins). Additional file 2:
**Phylogenetic tree of cnidarian bHLH transcription factors.** Maximum-likelihood and neighbour-joining analysis support orthology of cnidarian bHLH proteins used in this study. Additional file 3:
**Phylogenetic tree of FoxA transcription factors.** Maximum-likelihood and neighbour-joining analysis support orthology of cnidarian FoxA proteins). Additional file 4:
**Phylogenetic analysis of paired-class homeobox transcription factors.** Maximum-likelihood and neighbour-joining analysis support orthology of cnidarian paired-class proteins used in this study. Additional file 5:
**Phylogenetic analysis of BMP-related signalling factors.** Maximum-likelihood and neighbour-joining analysis support orthology of cnidarian BMP-related proteins used in this study. Additional file 6:
**Phylogenetic analysis of GATA transcription factors.** Maximum-likelihood and neighbour-joining analysis support orthology of cnidarian GATA factors. Additional file 7:
**Phylogenetic analysis of T-box transcription factors.** Maximum-likelihood and neighbour-joining analysis support orthology of cnidarian T-box proteins used in this study.

## Abbreviations

*bmp*: *bone morphogenetic protein*
bp: base pairs
DIG: digoxigenin
*Drgx*: *dorsal root ganglion homeobox*
EST: expressed sequence tag
F-actin: filamentous actin
*foxA*: *forkhead-box transcription factor A*
*otx*: *orthodenticle homeobox*
*otp*: *orthopedia homeobox*
PBS: phosphate buffered saline
PE: paired end
RQI: RNA quality indicator
RT: room temperature
*tbx*: *t-box*
*six*: *sine oculis*
