# Supplementary material for: Adoption of conserved developmental genes in development and origin of the medusa body plan
Source: EvoDevo. 2015 May 29;6:23. doi: 10.1186/s13227-015-0017-3 (PMC4464714; doi:10.1186/s13227-015-0017-3)
Supplement: Additional file 1: — Phylogenetic analysis of Six homeobox transcription factors. Maximum-likelihood and neighbour-joining analysis support orthology of cnidarian Six3 proteins). [file 13227_2015_17_MOESM1_ESM.docx]

**Additional file 1: Phylogenetic analysis of Six homeobox transcription factors**


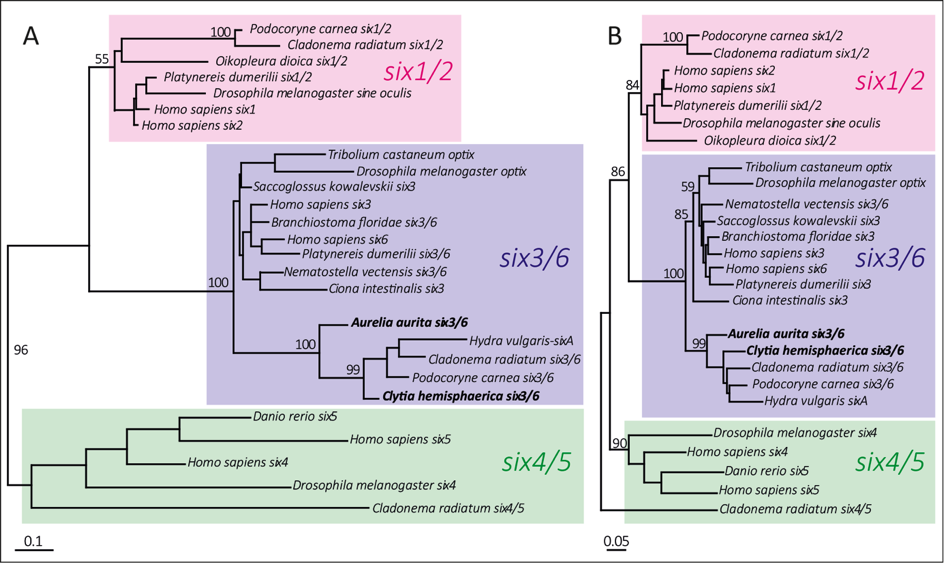


Gene orthology of SIX class homeobox transcription factors. **A**: Maximum-likelihood tree, **B**: Neighbour-joining tree. The medusozoan *six3/6* genes are grouping together within the *six3/6* family. Bootstrap values (in %) are placed next to the relevant nodes. Scale bars correspond to 0.1 or 0.05 changes per site, respectively.

Accession numbers of SIX proteins used:

Hs-six1 CAA62974.1, Cr-six1-2 AAT11873.1, Od-six1-2 AAZ23141.1, Pc_six1-2 AAT11871.1, Bf-six XP_002589159.1, Hs-six2 AAK16582.1|AF332197_1, Pd-six2 CAC86663.1, Pd-six3 CAR66435.1, Dm-sine AAF59260.1, Sk-six3 AAP79281.1, Hs-six3 AAD15753.1, Ci-six3 XP_002119543.2, Hs-six6 NP_031400.2, Dm-optix AAF59147.3, Tc-optix CAP58434.1, Nv-six3-6 AGD98926.1, Pc-six3-6 AAT11872.1, Cr-six3-6 AAT11874.1, Hv-sixA BAF56230.1, Hs-six4 BAA86223.1, Dm-six4 AAF51640.3, Cr-six4-5 AAT11875.1, Hs-six5 NP_787071.2, Dr-six5 NP_571795.1, Ch-six3-6 LN611635, Aa-six3-6 LN828925.
