# Supplementary material for: Adoption of conserved developmental genes in development and origin of the medusa body plan
Source: EvoDevo. 2015 May 29;6:23. doi: 10.1186/s13227-015-0017-3 (PMC4464714; doi:10.1186/s13227-015-0017-3)
Supplement: Additional file 2: — Phylogenetic tree of cnidarian bHLH transcription factors. Maximum-likelihood and neighbour-joining analysis support orthology of cnidarian bHLH proteins used in this study. [file 13227_2015_17_MOESM2_ESM.docx]

**Additional file 2: Phylogenetic tree of cnidarian bHLH transcription factors.**


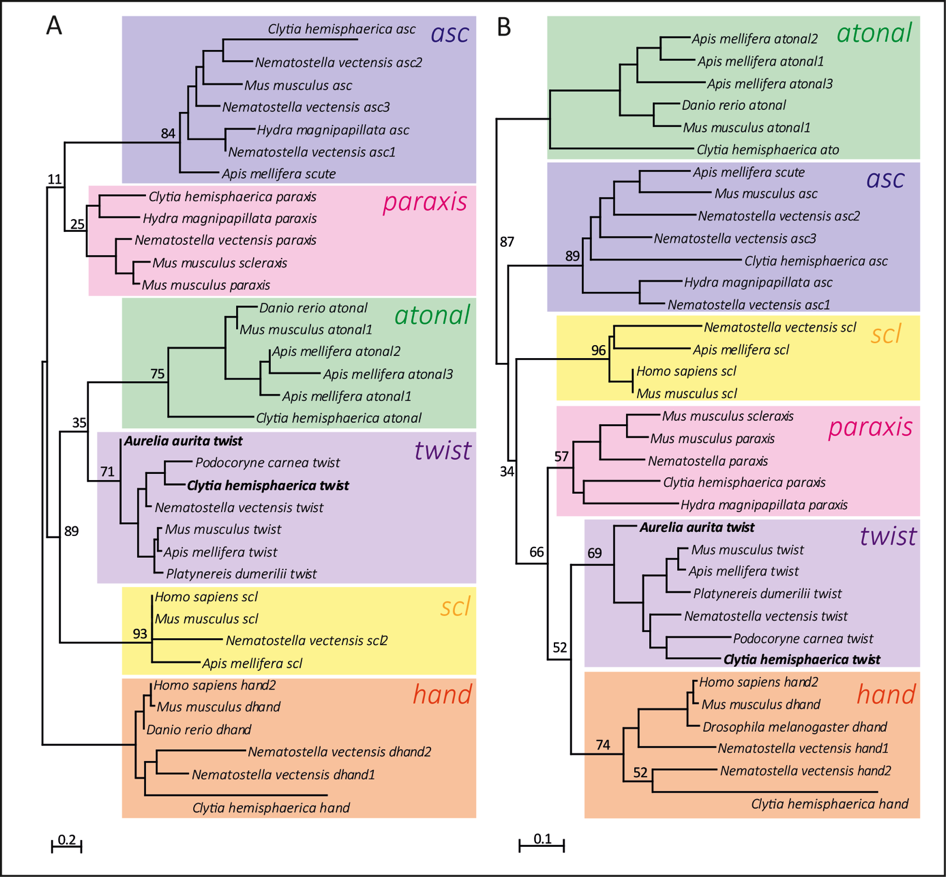


Gene orthology of cnidarian bHLH transcription factors. **A**: Maximum-likelihood tree, **B**: Neighbour-joining tree. Bootstrap values (in %) are placed next to relevant nodes. Scale bars correspond to 0.1 or 0.2 changes per site, respectively.

Accession numbers:

Mm-ato1 NP_031526.1, Mm-twi NP_035788.1, Mm-scl CAB72256.1, Mm-dhand CAC20671.1, Mm-paxis AAA86825.1, Pd-ato CAC86664.1, Dr-dhand AAF67130.1, Dr-ato NP_571166.1, Mm-paxis NP_942588.1, Dr-scl AAC41264.1, Hs-scl NP_003180.1, Hs-hand2 NP_068808.1, Hm-asc Hma1.121025:peptide, Am-scute GB18627-PA, Am-ato GB13897-PA, Am-ato2 GB15725-PA, Am-ato2 GB13095-PA, Mm-asc NP_032580.2, Am-dHand GB19031-PA, Am-scl GB13479-PA, Am-twi NP_001011637.1, Pd-twist AGQ04598.1, Pc-twist CAC12667.1, Nv-paraxis AFP87453.1, Mm-saxis NP_942588.1, Nv-dhand2 fgenesh1_pg.scaffold_80000048 80:425968-427027, Nv-scl fgenesh1_pg.scaffold_23000024 23:305463-306026, Nv-asc1 fgenesh1_pg.scaffold_370000011 370:159312-160680, Nv-asc2 fgenesh1_pg.scaffold_123000040 123:532534-533001, Nv-asc3 fgenesh1_pg.scaffold_81000007 81:74984-77632, Ch-paraxis LN611638, Ch-hand LN611640, Ch-asc LN611643, Ch-atl LN611644, Aa-twist LN611628, Ch-twist LN828923.
