# Supplementary material for: Adoption of conserved developmental genes in development and origin of the medusa body plan
Source: EvoDevo. 2015 May 29;6:23. doi: 10.1186/s13227-015-0017-3 (PMC4464714; doi:10.1186/s13227-015-0017-3)
Supplement: Additional file 3: — Phylogenetic tree of FoxA transcription factors. Maximum-likelihood and neighbour-joining analysis support orthology of cnidarian FoxA proteins). [file 13227_2015_17_MOESM3_ESM.docx]

**Additional file 3: Phylogenetic tree of FoxA transcription factors.**


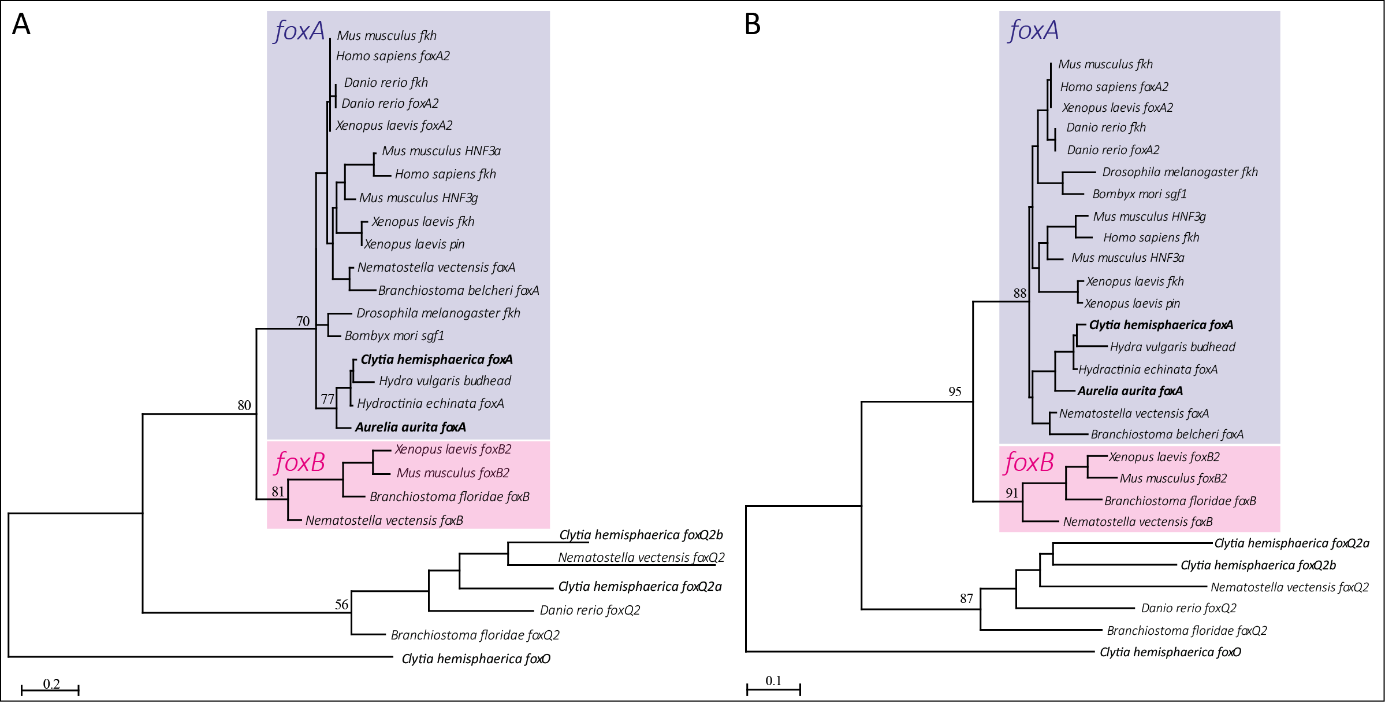


Gene orthology of *foxA* transcription factors. **A**: Maximum-likelihood tree, **B**: Neighbour-joining tree. Bootstrap-values (in %) are placed next to relevant nodes. Medusozoan *foxA2* genes cluster together while the ML tree (A) places *Nematostella foxA2* at the base of the *foxA2* genes. The NJ tree (B) places *Nematostella foxA2* next to *Branchiostoma foxA2*. Scale bars correspond to 0.1 or 0.05 changes per site, respectively.

Accession numbers:

Dr-foxA2 AAI65835.1, Dr-fkh NP_571024.1, Dm-fkh AAA28535.1, Hs-foxA2 AAH06545.2, Hsa-fkh AAA58477.1, Xl-foxA2 NP_001165629.1, He-foxA2 ADI82845.1, Bb-foxA2 ADD09805.1, Mm-foxB2 NP_032049.1, Xl-foxB2 CAD31848.1, Hv-budh AAO92606.1, Nv-foxA2 AAS13442.1, Bm-sgf1 BAA07523.1, Mm-fkh AAA03161.1, Xl-pin CAA46290.1, Xl-fkh AAB22027.1, Mm-HNF3g CAA52892.1, Mm-HNF3a CAA52890.1, Bf-FoxQ2 AAO23665.1, Dr-FoxQ2 NP_001098411.1, Nv-FoxQ2 AGD98927.1, Ch-FoxQ2b ABG21225.1, Ch-FoxQ2a ABG21224.1, Ch-FoxO ABG21226.1. Aa-foxA LN611630, ChFoxA LN611636.
