# Supplementary material for: Adoption of conserved developmental genes in development and origin of the medusa body plan
Source: EvoDevo. 2015 May 29;6:23. doi: 10.1186/s13227-015-0017-3 (PMC4464714; doi:10.1186/s13227-015-0017-3)
Supplement: Additional file 4: — Phylogenetic analysis of paired-class homeobox transcription factors. Maximum-likelihood and neighbour-joining analysis support orthology of cnidarian paired-class proteins used in this study. [file 13227_2015_17_MOESM4_ESM.docx]

**Additional file 4: Phylogenetic analysis of paired-class homeobox transcription factors.**


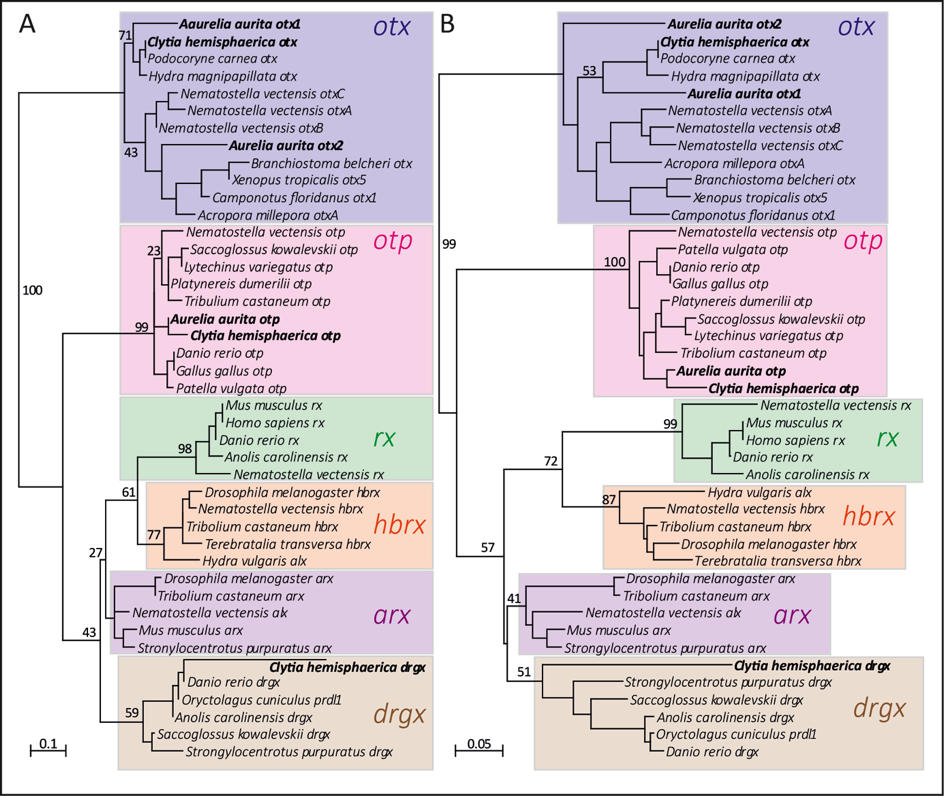


Gene orthology of medusozoan paired-class homeobox transcription factors. **A**: Maximum-likelihood tree, **B**: Neighbour-joining tree. Bootstrap-values (in %) are placed next to relevant nodes. Cnidarian *otx* and *otp* orthologs cluster together with bilaterian *otx* homologs. The gene published as *Hydra vulgaris aristaless*-like (*Hydra vulgaris alx*) is most likely a *homeobrain* homolog. *Clytia hemisphaerica drgx*-like clusters together with bilaterian *drgx* genes but seems to be rather derived (long branch). Scale bars correspond to 0.1 or 0.05 changes per site, respectively.

Accession numbers of proteins used:

Pv-otp AAM33145.1, Sk-otp NP_001158374.1, Nv-otp ADG03433.1, Pd-otp ABR68849.1, Tc-otp NP_001163995.1, Pc-otx AAF04002.1|AF160992_1, Cf-otx1 EFN64249.1, Am-otxA ABK41270.1, Lv-otp 74837540|sp|Q6SZ65.1, Gg-otp XP_003643004.1, Dr-otp XP_005170306.1, Nv-otxA ACO53861.1, Nv-otxB ACO53862.1, Hm-otx XP_002161581.1, Xt-otx5 NP_001016021.1, Bb-otx ACR66219.1, Sp-drgx XP_003729834.1, Nv_otxC AFJ11252.1, Oc-Prdl1 XP_002718385.1, Dr-drgx NP_001032182.1, Sk-drgx XP_002735289.1, Ac-drgx XP_003225456.1, Tc-arx NP_001107838.1, Dm-arx AAF51505.1, Mm-arx NP_031518.2, Sp-arx XP_791442.1, Hs-rx NP_038463.2, Dr-rx AAB62325.2, Mm-rx AAB62324.1, Ac-rx XP_005105348.1, Nv-hbrx ADG03434.1, Dm-hbrx NP_788420.1, Tc-hbrx EFA07441.1, Tt-hbrx AEZ03833.1, Hv-alx AF295531_1, Nv-alx scaffold_365:176934-179107, Nv-rx scaffold_62:785716-790320, Ch-drgx LN611639, Ch-otp LN611641, Ch-otx LN611646, Aa-otx1 LN611633, Aa-otx2 LN611634, Aa-otp LN611632,
