# Supplementary material for: Adoption of conserved developmental genes in development and origin of the medusa body plan
Source: EvoDevo. 2015 May 29;6:23. doi: 10.1186/s13227-015-0017-3 (PMC4464714; doi:10.1186/s13227-015-0017-3)
Supplement: Additional file 5: — Phylogenetic analysis of BMP-related signalling factors. Maximum-likelihood and neighbour-joining analysis support orthology of cnidarian BMP-related proteins used in this study. [file 13227_2015_17_MOESM5_ESM.docx]

**Additional file 5: Phylogenetic analysis of BMP-related signaling factors.**


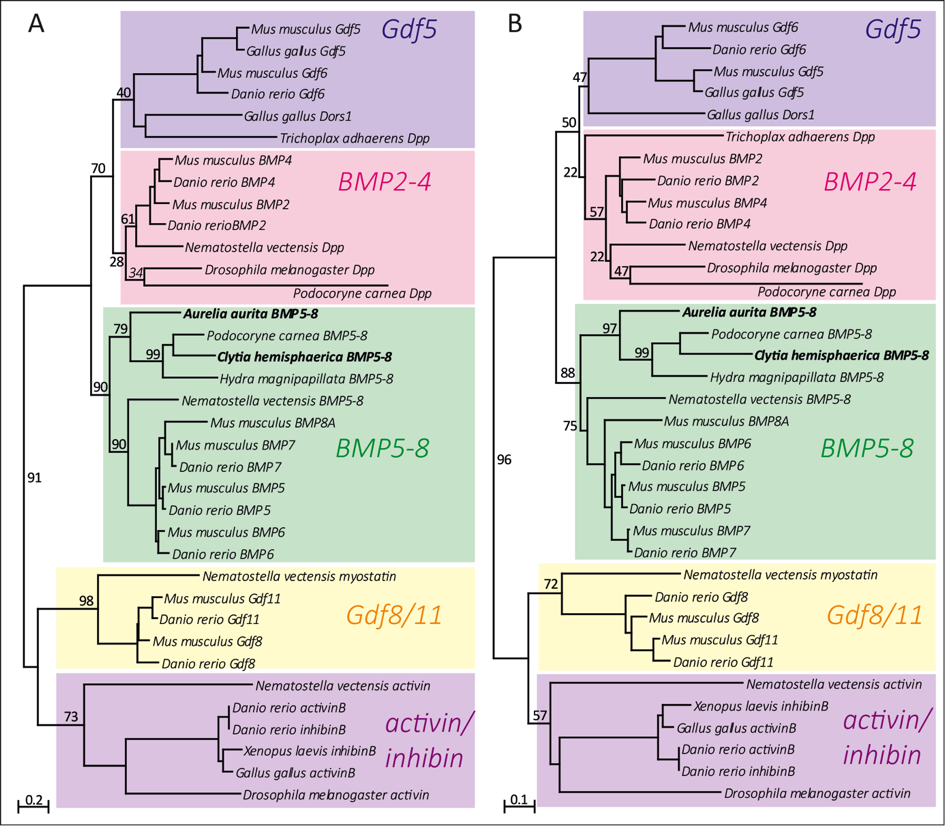


Gene orthology trees of BMP/Tgfß proteins. **A**: Maximum-likelihood tree. **B**: Neighbour-joining tree. Bootstrap-values (in %) are placed next to relevant nodes. Scale bars correspond to 0.1 or 0.2 changes per site, respectively.

Accession numbers of proteins used:

Nv-activin ABF61781.1, Nv-myostain AGL96595, Dr-GDF11 NP_998140.1, Mm-GDF11 NP_034402.1, Dr-GDF8 NP_571094.1, Mm-GDF8 NP_034964.1, Dm-activin AAL51005.1, Dr-Inhibinβ CAQ13345.1, Dr-Activinβ CAA53636.1, Gg-Activinβ CAA96248.1, Xl-Inhibinβ AAH77857.1, Gg-Gdf5 NP_989669.1, Mm-GDF5 NP_032135.2, Dr-GDF6 NP_571062.1, Mm-GDF6 NP_038554.1, Nv-BMP 5-8 ABC88372.1, Dr-BMP7 NP_001070614.1, Mm-BMP7 NP_031583.2, Pc-BMP5-8 ABA42602.1, Dr-BMP5 NP_957345.1, Mm-BMP5 NP_031581.2, Dr-BMP6 NP_001013357.1, Mm-BMP6 NP_038554.1, Mm-BMP8a NP_001242948.1, Nv-Dpp AAR27580.1, Pc-Dpp ABA42601.1, Dm-Dpp NP_477311.1, Dr-BMP4 NP_571417.1, Mm-BMP4 NP_031580.2, Mm-BMP2 NP_031579.2, Dr-BMP2 NP_571435.1, Pc-BMP5-8 ABA42602.1, Hm-BMP5-8 XP_002165935.2, Aa-BMP5-8 AGN03874.1, Ta-Bmp2-4 XP_002113173.1, Gg-dorsalin1 NP_990763.1, Ch-BMP5-8 LN611637
