# Supplementary material for: Adoption of conserved developmental genes in development and origin of the medusa body plan
Source: EvoDevo. 2015 May 29;6:23. doi: 10.1186/s13227-015-0017-3 (PMC4464714; doi:10.1186/s13227-015-0017-3)
Supplement: Additional file 6: — Phylogenetic analysis of GATA transcription factors. Maximum-likelihood and neighbour-joining analysis support orthology of cnidarian GATA factors. [file 13227_2015_17_MOESM6_ESM.docx]

**Additional file 7: Phylogenetic analysis of GATA transcription factors.**


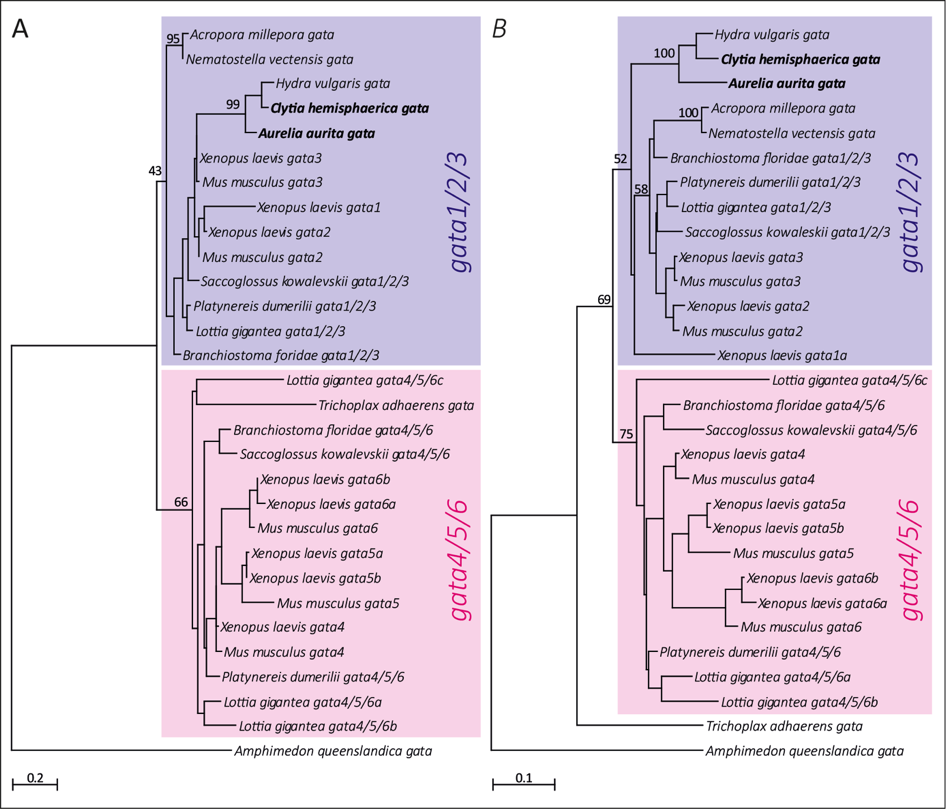


Gene orthology trees for *gata*-genes shown in the present work. Medusozoan *gata*-homologs cluster together and loosely group with the bilaterian *gata1/2/3* family. **A**: Maximum-likelihood tree. **B**: Neighbour-joining tree. Bootstrap-values (in %) are placed next to relevant nodes. Scale bars correspond to 0.1 or 0.2 changes per site, respectively.

Accession numbers of GATA proteins:

Bf-gata1/2/3 ACR66214.1, Bf-gata4/5/6 ACR66216.1, Sk-gata4/5/6 NP_001164701.1, Sk-gata1/2/3 NP_001161551.1, Ac-gata aug_v2a.16813.t1 aug_v2a.16813 scaf8056:40646-45844(-), Hv-gata Hma1.123787: peptide, Nv-gata AAR24452.1, Pd-gata1/2/3 ABK32792.1, Xl-gata1 AAI70023.1, Xl-gata2 NP_001084043.1, X-gata3 AAA49724.1, Xl-gata4 NP_001084098.1, Xl-gata5a NP_001081962.1, Xl-gata5b AAH55963.1, Xl-gata6a NP_001081452.1, Xl-gata6b NP_001083725.1, Mm-gata2 AAI07011.2, Mm-gata-3 NP_032117.1, Mm-gata4 NP_032118.2, Mm-gata6 AAD55267.1|AF179425_1, Ta-gata XP_002115729.1, Aq-gata Aqu1.223181, Mm-gata5 AAI05655.1, Lg-gata4/5/6a: e_gw1.67.75.1, prot. ID: 129788, Aa-gata LN611645, Ch-gata LN61162.
