# Supplementary material for: Adoption of conserved developmental genes in development and origin of the medusa body plan
Source: EvoDevo. 2015 May 29;6:23. doi: 10.1186/s13227-015-0017-3 (PMC4464714; doi:10.1186/s13227-015-0017-3)
Supplement: Additional file 7: — Phylogenetic analysis of T-box transcription factors. Maximum-likelihood and neighbour-joining analysis support orthology of cnidarian T-box proteins used in this study. [file 13227_2015_17_MOESM7_ESM.docx]

**Additional file 7: Phylogenetic analysis of T-box transcription factors.**


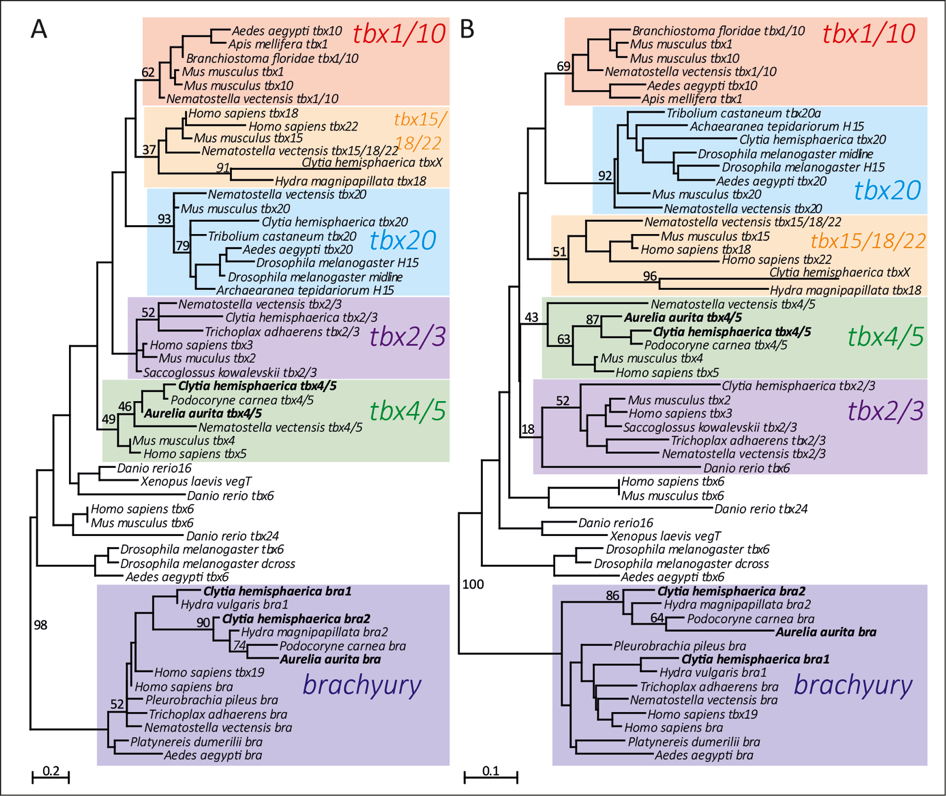


Gene orthology trees of T-box transcription factors shown in the present work. **A**: Maximum-likelihood tree **B**: Neighbour-joining tree. Bootstrap-values (in %) are placed next to relevant nodes. Scale bars correspond to 0.1 or 0.2 changes per site, respectively.

Accession numbers of T-box proteins:

Nv-tbx15 estExt_gwp.C_190152, Hs-tbx18 AAH40697.2, Mm-tbx15 O70306, Mm-tbx2 NP_033350.2, Hs-tbx3 AAC12947.1, Mm-tbx10 NP_001001320.1, Nv-tbx1-1 AAQ23383.1, Mm-tbx1 P70323, Bf-tbx1-10 AAG34887.2, Nvtbx2/3 estExt_gwp.C_650148, Ta-tbx2-3 CAD70270.1, Am-tbx1 XP_624689.2, Dm-midline NP_608927.2; Aae-tbx20 EAT39897.1, Aae-tbx6 EAT44470.1, Nv-tbx5-1 estExt_gwp.C_650150, Mm-tbx20 NP_065242.1, Tc-tbx20a XP_972626.1, Pc-tbx4-5 CAE45765.1, Pc-bra CAD21521.1, Hs-tbx6 AAH26031.1, Mm-tbx6 AAC53110.1, Dr-16 NP_571133.1, Pp-bra CAE45766.1, Pd-bra CAC19335.1, Hs-bra CAA04938.1,Ta-bra CAD70269.1, Aae-bra EAT35953.1, Hs-tbx22 CAI43070.1, Nv-tbx20-4 gw.146.32.1, At-H15 BAD16721.1, Dm-H15 CAA67304.1, Xla-vegT AAB93301.1, Hs-tbx18 CAB37937.1, Dr-tbx24 BAB97199.1, Mm-tbx4 NP_035666.1, Hma-tbx18 XP_004206516.1, Aae-tbx10 XP_001659532.1, Sk-tbx2-3 NP_001158392.1, Hs-tbx5 AAC51644.1, Dr-tbx6 NP_571127.1, Dm-dcross NP_648283.1, Hs-tbx19 NP_005140.1, Nv-bra AAO27886.2, Hv-bra1 AAD26626.1, Hma-bra2 Hma1.135445, Ch-bra1 ABJ16449.1, Ch-bra2 JAC85032.1, Aa-bra LN611629, Aa-tbx4-5 LN611631, Ch-tbx2-3 LN611642, Ch-tbx4-5 LN828922, Ch-tbx20 LN828924.
